# The Process of Filopodia Induction during HPV Infection

**DOI:** 10.3390/v14061150

**Published:** 2022-05-26

**Authors:** Alyssa Biondo, Patricio I. Meneses

**Affiliations:** Department of Biological Sciences, Fordham University, Bronx, NY 10458, USA; abiondo4@fordham.edu

**Keywords:** HPV16, filopodia, viral entry

## Abstract

Human Papillomavirus 16 (HPV16) infects mucosal and epithelial cells and has been identified as a high-risk HPV type that is an etiologic agent of human cancers. The initial infectious process, i.e., the binding of the virus particle and its entry into the host cell, has been studied extensively, although it is not fully understood. There is still a gap in understanding the steps by which the virus is able to cross the plasma membrane after receptor binding. In this study, we demonstrate that after HPV16 comes into contact with a plasma membrane receptor, there are cytoskeletal changes resulting in an increase of filopodia numbers. This increase in filopodia numbers was transient and was maintained during the first two hours after virus addition. Our data show that there is a statistically significant increase in infection when filopodia numbers are increased by the addition of drug and virus simultaneously, and a decrease in virus infection when filopodia formation is inhibited. We describe that HPV16 binding results in the activation of Cdc42 GTPase that in turn results in an increase in filopodia. siRNA directed at Cdc42 GTPase resulted in a statistically significant reduction of infection and a corresponding lack of filopodia induction.

## 1. Introduction

*Papillomaviridae* is a family of non-enveloped DNA viruses that infect squamous epithelial tissues [1,2]. The HPV viral capsid is composed of the L1 and the L2 viral proteins [1]. The capsid shell protects a circular, double stranded chromatinized DNA genome of approximately 8,000 base pairs. HPV infections are common in sexually active individuals, and although most infections are cleared within a few months, some infections remain latent and lead to diseases including cancers [3]. Our research focuses on HPV16, a high-risk type responsible for 50% of cervical cancer cases and a growing number of oropharyngeal cancers [2]. The events leading to the endocytosis of the HPV16 virus is still unclear. We hypothesize that virus binding to a plasma membrane receptor starts a signal cascade that induces filopodia formation. By increasing the number of filopodia there is a resulting increase in infection. 

Multiple studies have indicated HPV16′s reliance on actin rearrangement during infection [4,5,6]. We have shown that inhibition of the nonreceptor tyrosine kinase FAK/PYK2 family prevents filopodia formation and results in a loss of virus infection [7,8]. Inhibition of the PI3K/Akt/mTOR pathway by Surviladze and colleagues resulted in a loss of HPV16 infection [6]. PI3K/Akt/mTOR pathways are associated with modulating actin rearrangement pathways [6,9]. Actin depolymerization using cytochalasin D inhibited HPV16 infection in HaCaT cells [5]. In the latter publication, cells treated with cytochalasin D appeared to have congregated irregular vesicles with virions arrested at the cell membrane, and the authors suggested that HPV16 relied on the actin rearrangement for vesicle scission and macropinocytosis [5]. We hypothesize that the described lack of viral entry and trafficking events in these studies is in part due to a loss of filopodia formation. Filopodia are induced and are often exploited by enveloped and naked viruses to enter their target cells including HIV, HSV, VSV, and HPV31 [10]. 

Filopodia are membrane protrusions used by all cell types to probe the extracellular environment, facilitate cell adhesions with the extracellular matrix (ECM), participate in cell migration, and establish cell-cell contacts during wound healing [10,11]. Filopodia’s innate role in cellular processes helps contribute to viral infection by: (1) increasing the surface area for viruses to bind to target cells, (2) interacting with neighboring cells to form bridges for viruses to move from one infected cell to an uninfected cell, and (3) allow viruses to bind and move towards the cell body through actin-retrograde movement [10,11]. All these events are mediated by a membrane bound receptor in the case of HPV16, as is the case with HSV, HHV8, HIV, and VSV, and this is most likely a glycoprotein [10].

In this study we describe that initial binding of viral particles to the plasma membrane results in filopodia formation. We quantify these filopodia after the addition of the HPV16 pseudovirions and demonstrate how filopodia changes affect infection. We use chemical induction and interference of filopodia formation to show that there is a positive correlation between the increase in filopodia number and percent infection. In addition, we identify that a GTPase involved in cytoskeletal rearrangement is activated upon virus binding to the plasma membrane and use siRNA to show that the lack of these cytoskeletal changes results in a loss of infection. Taken together, our data suggest that HPV16 stimulates filopodia formation via the Cdc42 GTPase signaling pathway. Our data helps to fill the gap in the HPV field in understanding the relationship between virus binding and host cytoskeleton rearrangement. 

## 2. Materials and Methods

### 2.1. Cells and HPV16 PsV Production

HaCaT cells (spontaneously immortalized keratinocyte cells) purchased from AddexBio (San Diego, CA, USA) were cultured in Dulbecco’s Modified Eagle’s Media (DMEM) (Thermo Scientific, Somerset, NJ, USA) supplemented with 10% Fetal Bovine Serum [FBS, (Gemini Bio-Products, West Sacramento, CA, USA)]. HPV16 pseudovirions (PsVs) were produced in 293TT cells (human embryonic kidney cells) as described by Buck et al. [12]. In brief, the p8fwb plasmid was used for the PsV pseudogenome encoding reporter gene GFP. The p16sheLL encodes humanized HPV16 L1 and L2 gene products. Both 8fwb and p16sheLL were co-transfected into 293TT cells with GenJet transfection reagent (Signagen, Ballenger Creek, MD, USA). 293TT cells were a generous gift from Dr. Ozbun (The University of New Mexico School of Medicine, Albuquerque, NM, USA). Two days post transfection, cells were harvested with a lysis buffer (PBS, triton-X 10%, benzonase, plasmid-safe). Cell lysates were incubated overnight at 37 °C to mature assembled PsVs. PsVs were purified from cell lysates using an optiprep density gradient medium in PBS: 27%, 32%, 37% (Sigma, St. Louis, MO, USA). The percentage of infectivity was determined via flow cytometry (BD Accuri C6, Franklin Lakes, NJ, USA) by measuring the number of GFP positive HaCaT cells after 48 h. Our experiments were performed with approximately 800 viral genome equivalents (VGE) per cell. VGE was determined by quantitative PCR of p8fwb with the StepOne real-time PCR system (ThermoFisherScientific, Waltham, MA, USA).

### 2.2. Flow Cytometry

Cell media was changed and replaced with serum free DMEM 30 min prior to PsV addition. Cell plates were incubated on ice for 2 h after viral addition. Unbound virus was removed from cells and wells were washed three times with 1× PBS. DMEM supplemented with 10% FBS was added to each well. Plates were incubated for 48 h and then cells were harvested with 0.05% trypsin (Thermofisher, Waltham, MA, USA). Cells were washed three times with 1× PBS and pelleted by centrifugation for 5 min at 3000 rpm. Samples were resuspended in 100 µL of PBS and 10,000 events in the desired cell population were counted.

### 2.3. Number of HaCaT Cells Seeded for Experiments

Flow cytometry: Cells were plated at 50,000 per well in a 24 well plate.

Immunofluorescent slides: Cells were plated at 50,000 per well in a 12 well plate containing coverslips. 

Drug treatments: Cells were plated at 200,000 per well and incubated for three days in a six well plate.

Cdc42 activation pulldown: Cells were plated at 200,000 per well and incubated for two days in a six well plate. 

Cdc42 siRNA experiments: Cells were plated at 75,000 per well in a 12 well plate.

All cells were grown in an incubator with CO_2_ levels set at 5% and temperature at 37 °C. 

### 2.4. Immunofluorescence Microscopy

Cells were imaged with a Leica TCS SP8 confocal microscope (Leica Microsystems; Wetzlar, Germany) using a 63× oil immersion objective lens. Z stack intervals were taken from the top to bottom of the cells in 0.20 µm intervals. 

Cells were fixed on coverslips with 4% paraformaldehyde (PFA) in PBS for 15 min on ice and permeabilized and blocked for 30 min with a blocking buffer (0.02% Triton-X 100, 0.02% Fish Gelatin, 1× PBS). Coverslips were incubated with primary antibodies at 1:100 for 1 h and then secondary antibodies at 1:2000 for 30 min in blocking buffer. Coverslips were washed three times with 1× PBS before and after primary and secondary antibody incubations. Alexa Fluor 488 phalloidin (Life Technologies, Carlsbad, CA, USA) was used to stain F-actin and was diluted 1:100 with secondary antibodies in blocking buffer. Coverslips were mounted on slides with ProLong^®^ Gold Antifade Mountant with the addition of 4′,6-diamidino-2-phenylindole (DAPI, Life Technologies) to visualize cell nuclei. 

Primary antibodies used for immunofluorescence included: EEA1 antibody for early endosome (C-15, sc-6414, Santa Cruz Biotechnologies, Dallas, TX, USA), H16.V5 antibody for HPV16 L1 (a kind gift from Dr. Neil Christensen, Penn State University, Hershey, PA, USA). Secondary antibodies were tagged with Alexa Fluor dyes from Life Technologies: donkey anti-rabbit 594, donkey anti-sheep 594, donkey anti-goat 594, and donkey anti-mouse 647.

### 2.5. Infection Experiments

Cells were plated on coverslips two days prior to the experiment and incubated with cold DMEM serum free media on ice for 30 min prior to PsV infection. A review of the literature showed that infection experiments with HPV16 are used at 500–5000 VGEs, accordingly, we conducted our experiments at 800 VGEs per cell for IF experiments [4,5]. Using real-time PCR, we determined the number of genomes in our preparation to be 800 DNA containing viral particles per cell using a MOI of 0.15. L1 protein in our viral preparation was determined by comparing L1 levels to control amounts of BSA in a Coomassie stained gel. We measured 30 million viral capsids in a 0.15 MOI or 7500 viral particles per cell/experiment. After PsV addition, HaCaT cells were incubated for 5 min, 15 min, 30 min, 1 h, 2 h, 4 h, 6 h, and 8 h at 37 °C. Time 0 min was used as a control, where the virus was added and cells were immediately harvested. Unbound PsVs were removed from wells after all infection time points with three 1× PBS washes. Cells were fixed for immunofluorescence with 4% PFA for 15 min on ice. Filopodia were visualized with phalloidin staining. The number of filopodia was the average count in fifteen cells at each timepoint in three separate experiments (*n* = 45) using Las X Life Science Microscope Software Platform.

### 2.6. Filopodia Number Analysis

Optical sections of cells were imaged at 0.20 µm intervals from the bottom of the cell using a TSP5 confocal microscopy system. For each image, 15–20 optical sections were taken, and the max projection of the stack was used for analysis. Filopodia were counted manually in the Leica program and measured with the micro-ruler tool in the Leica program. Filopodia numbers and lengths were also evaluated using the FiloQuant plugin for Image J software. A region of interest was drawn around one cell based on the presence of nuclei. Filopodia were only quantified along the periphery of cells bordering a gap. We did not quantify filopodia of cells that are present ventrally or dorsally and between cells. Single image analysis detected the number of filopodia and their lengths at the cell boundary. Filopodia was defined as >1 µm. The analysis of filopodia relied on intensity based thresholding, which is described in detail by Guilaume Jacquemet and colleagues [13]. FiloQuant determines filopodia numbers and lengths by generating an intensity-based threshold of the cell edge that is free of filopodia protrusions. The original image is then enhanced to optimize filopodia detection. The two images are overlayed to detect filopodia which are then counted and outlined using the skeletonized image J plugin as shown in Supplementary Figure S1. 

### 2.7. Drug Treatment Preparation

For immunofluorescence experiments, cells were serum starved for 8 h prior to drug treatment. Concentrations of 200 ng/mL bradykinin (0.188 µM), 200 ng/mL EGF (0.312 mM), and 10 µM ML-141 were prepared in DMEM serum free media. HaCaT cells were treated with either bradykinin and EGF for 15 min, or 2 h with ML-141.The virus was added simultaneously with the drug treatment. Bradykinin acetate salt was purchased from Sigma (Cat. 90834-10MG), epidermal growth factor (EGF) was purchased from Fisher Scientific (Cat. 236EG200), and ML-141 was purchased from Tocris (Cat. No. 4266). 

For flow cytometry experiments, cells were serum starved for 4 h prior to the addition of drug treatment. Cells were treated with 10 µM ML-141 for 2 h prior to viral addition to inhibit filopodia. Bradykinin and EGF were added with virus simultaneously for flow cytometry experiments. Fresh drug was replaced every 2 h for 8 h. After 8 h, cells were washed with 1× PBS, and DMEM supplemented with FBS was added. ML-141 was effective in inhibiting filopodia formation after 2 h of incubation but lost the effect after 4 h. ML-141 was replaced every two hours to ensure that we did not see any filopodia. Cells were incubated with DMEM supplemented with FBS for two days before flow cytometry experiments were performed. For all flow cytometry experiments we also performed immunofluorescence to count the filopodia of cells that were treated identically to the samples that were used for flow.

### 2.8. Western Blots

The primary antibodies were used in western blot assays at 1:1000 dilution in a TNET wash buffer (1 M Tris pH 7.5, 0.5 M EDTA, NaCl in dh_2_O). Antibodies: Cdc42 (ab187643, Abcam, Cambridge, United Kingdom), EEA-1 for early endosome (sc-6414, Santa Cruz Biotechnology, Dallas, TX), actin antibody (A3853; Sigma-Aldrich, St. Louis, MO, USA), and Anti-HPV16 L1 (MD2H11, Santa Cruz Biotechnology, Dallas, TX, USA). IRDye secondary antibodies (anti-rabbit and anti-mouse 680 and 800) from Li-Cor (Lincoln, NE, USA) were used at 1:30,000 and 1:20,000 respectively. Cell lysates were harvested in Nonidet P-40 lysis buffer [0.05% NP40, 0.86% NaCl, 0.5 M EDTA, Tris Base in dH_2_O and supplemented with Halt protease/phosphatase inhibitor cocktail (Life Technologies, Carlsbad, CA, USA)]. 

Samples were subjected to SDS-PAGE gel electrophoresis and transferred to nitrocellulose membrane (ThermoFisher Scientific, Waltham, MA, USA). Membranes were blocked for 1 h in 5% BSA in TNET, and incubated in primary antibodies either overnight at 4 °C or for 4 h at room temperature. Secondary antibody incubations were carried out for 45 min at room temperature. Membranes were washed three times after each antibody incubation with 1× TNET. Proteins were visualized and band intensities measured on the Li-Cor Odyssey Imaging System. Actin protein levels were used for normalization in western blot assays.

### 2.9. Colocalization

Colocalization was evaluated with JaCoP plugin for imageJ. Mander’s coefficient M2 calculated the fraction of overlap between the virus signal and EEA1 signal. The H16.V5 signal was pseudocolored red and the EEA1 signal was psuedocolored green. Early endosome and HPV16 L1 capsid overlap were used as a marker for virus internalization. Early endosome was used as a marker for internalization because this is one of the first organelles the virus is trafficked to after entry. Thresholds were set manually and constant for every scan. Z stack intervals were kept consistent for every experiment from top to bottom in 0.20 µm intervals. Analysis was performed for the whole Z-stack. Data shown is from one of the six separate experiments.

### 2.10. siRNA Transfections

Accell siRNA SMART pools for Cdc42 were purchased from Origene (Montgomery, MA, USA). Non-targeting control siRNA was purchased from IDT (Coralville, IA, USA). RNAi Max Lipofectamine was purchased from Life Technologies (Norwalk, CT, USA). SiRNA knockdowns was performed using 100 pmoles siRNA for 48 h at 37 °C.

### 2.11. Active Cdc42 Pull-Down Assay and Glutathione PAK-PBD Coated Beads

PsVs or an optiprep gradient used to separate viral fractions (control) were added to wells and incubated at 37 °C for each timepoint. Unbound virus was removed from wells with three washes of cold 1× PBS. Cells were harvested from wells with lysis buffer (50 mM Tris pH 7.5, 10 mM MgCl_2_, 0.3 M NaCl, 2% IGEPAL in PBS) and transferred to cold Eppendorf tubes. Samples were centrifuged for 5 min at 10,000 rpm. Cell pellets were discarded, and the supernatant was assessed for protein concentration. Cell lysates were diluted to 500 µg/uL of total protein with lysis buffer. 

Bacterial subcultures of pGEXTK-Pak-1 70-117 (addgene, #12217) were grown to an optical density of 0.5 and induced with 0.05 mM IPTG for 2.5 h at 37 °C. Bacteria cells were pelleted and harvested with lysis buffer (0.5 M EDTA, Tris 7.5 pH, lysozyme, DTT, Halt™ protease and phosphatase inhibitor in 1× PBS). Halt™ protease and phosphatase cocktail (100×) inhibited phosphatases and proteases targeting serine/threonine proteins and was diluted to 1× in solution. Bacteria lysates were rotated and incubated with glutathione beads overnight at 4 °C. Beads were washed three times with immunoprecipitation wash buffer (10 mM Tris pH 7.4, 1 mM EDTA, 150 mM NaCl, 1% Triton X-100, and 0.2 mM sodium orthro-vandadate) as described by Leinco Technologies. HaCaT cell lysates were incubated with 15 µg of PAK-PBD beads and rotated at 4 °C for 1 h. Samples were washed three times with GTPase wash buffer (25 mM Tris pH 7.5, 30 mM MgCl_2_, 40 mM NaCl) and then loaded with 1× laemmli dye for gel electrophoresis. Control samples were treated with 200 µM GTPỿ and 10 mM GDP. GTPỿ and GDP were added to samples with 150 mM EDTA and were rotated for 15 min at room temperature. GTPỿ and GDP binding was stopped with 600 mM MgCl_2_. PAK-PBD beads were then added to controls incubated with GTPỿ and GDP for 1 h with rotation. 

### 2.12. Statistical Analysis

All ANOVA Dunnett’s multiple comparison tests of variance were performed with Graphpad Prism software v. 8 (Graph Pad, San Diego, CA, USA). A *p* < 0.05 was considered a statistically significant difference between samples. A statistical analysis was performed and graphed using Graphpad Prism software. 

### 2.13. Propidium Iodine Cell Cycle Staining

HaCaT cells were plated at 500,000 and transfected with control or Cdc42-siRNA the next day. The virus was added to wells after 24 h of Cdc42 siRNA knockdown. Cells were harvested and pelleted at 300× *g* for 7 min. Cells were resuspended in cold 1× PBS two times. In a separate tube, 2 × 10^6^ cells were transferred and vortexed slowly as 1 mL of cold 100% ethanol was added. Cells were fixed at 4 °C for 1 h and then centrifuged for 6 min at 1000× *g*. Cells were resuspended in Triton-X/PI solution (0.1% Triton-X 100, 0.2 µg/µL of RNase A, 5 µg/mL in 1× PBS). Cells were rotated at 37 °C for fifteen minutes and then analyzed with flow cytometry. 

## 3. Results

### 3.1. HPV16 PsV Addition Induces an Increase in the Number of Filopodia on HaCaT Cell Membranes

We had previously published an observation showing an increase in filopodia after the addition of HPV16 pseudovirions and that the FAK inhibitor TAE226 prevented the formation of filopodia and viral infection was reduced [8]. Here we addressed if there was a direct relationship between virus binding, time of infection, and filopodia formation.

**Figure 1:**
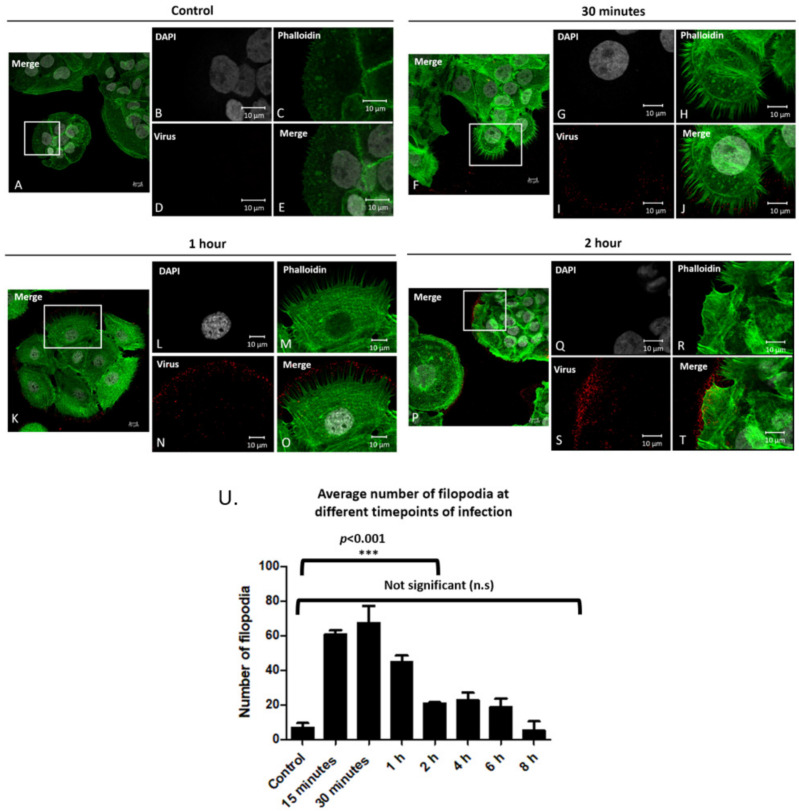
Filopodia numbers increase after the addition of PsVs. Confocal microscopy images of control cells without PsVs addition (**A**–**E**), and after PsV addition for 30 min (**F**–**J**), 1 h (**K**–**O**), and 2 h (**P**–**T**). Cell nuclei were stained with DAPI (grey), phalloidin stained filopodia (green), and H16.V5 antibody stained HPV16 L1 protein (red). Full merged images of all channels (**A**,**F**,**K**,**P**). Zoomed in images of merged images (**E**,**J**,**O**,**T**). Average number of filopodia per cell after viral addition for 15 min–8 h (**U**). Filopodia were counted using LAS X Life Science Microscope Software Platform for fifteen cells at each timepoint for three separate experiments (*n* = 45). Images are shown in X, Y, Z planed Z stacks. An ANOVA Dunnett’s multiple comparisons test was used to evaluate statistical significance (samples were compared to control cells, ***, *p* < 0.001). Averages are displayed with SEM.

We visualized the filopodia on the periphery of uninfected cells, and cells infected for 30 min, 1 h and 2 h after virus addition (Figure 1A–E, Figure 1F–J, Figure 1K–O, Figure 1P–T respectively). Filopodia were quantified using the FiloQuant image J plugin as shown in Figure S1. Images show an increase in filopodia after 30 min that did not change after 1 h but did decrease after 2 h. Data showed that there is staining of virus particles (stained in red) that does not coincide with the plasma membrane visualized by F-actin staining with phalloidin (stained in green). We show that this non-plasma membrane staining overlaps with the pattern of laminin-5 in the extracellular matrix (Supplementary Figure S2). Figure S3 has images of cells at 4, 6, and 8 h.

The average number of filopodia were: 7 in control cells, and 61, 67, 45, 21, 23, 19, 5 after 15 min, 30 min, 1 h, 2 h, 4 h, 6 h, and 8 h post infection, respectively (Figure 1U). 

Data in Figure 1Q represents the averages of filopodia for 15 cells for three separate experiments (*n* = 45). These data show a statistically significant increase in filopodia in the first hour after the addition of viral particles (Figure 1U, ***, *p* < 0.001). 

### 3.2. Bradykinin and EGF Induce an Increase in the Number of Filopodia

To test if the number of filopodia influenced the level of viral infection, we tested two filopodia inducing drugs. We used bradykinin and epidermal growth factor (EGF), two well-established wound healing mediators that aid in filopodia formation [14,15,16,17,18]. 

**Figure 2:**
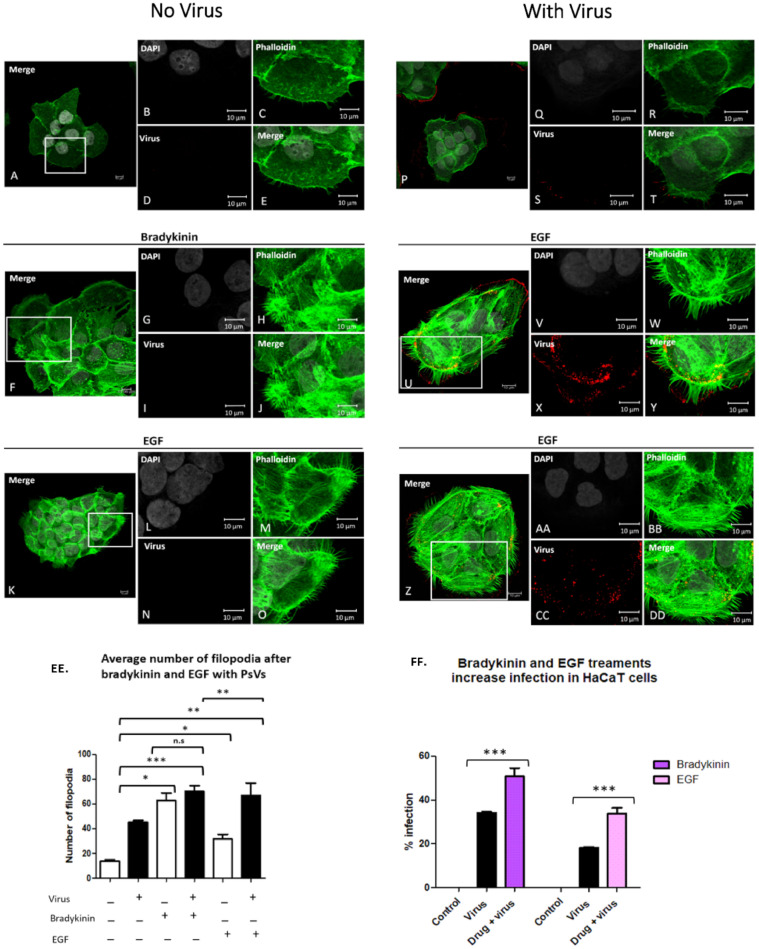
Bradykinin and EGF treatments increase the average number of filopodia in HaCaT cells and increase infection. HaCaT cells were seeded onto coverslips and treated with 200 ng/mL bradykinin, 200 ng/mL EGF, plus virus for 15 min. Confocal microscopy images of control cells (**A**–**E**), and cells treated with bradykinin (**F**–**J**) or EGF (**K**–**O**) without virus. Cells incubated with virus alone (**P**–**T**) or with virus and bradykinin (**U**–**Y**) or EGF (**Z**–**DD**). Nuclei stained with DAPI (grey), filopodia visualized with phalloidin (green), and L1 capsid stained with H16.V5 antibody (red). All channels were merged (**E**,**J**,**O**,**T**,**Y**,**DD**). Graph representation of average filopodia numbers for control cells and cells treated with virus, bradykinin, EGF, or drug with virus (**EE**). Filopodia were counted using LAS X Life Science Microscope Software Platform. Statistical significance was determined by ANOVA Dunnett’s multiple comparison test (*n* = 45, *, *p* < 0.05, **, *p* < 0.01, ***, *p* < 0.001). Percent infection with or without drug treatment was measured with flow cytometry (**FF**). Flow cytometry data of infection in HaCaT cells with or without bradykinin and EGF. Data was taken from three individual experiments in triplicate. Statistical differences determined by ANOVA Dunnett’s multiple comparison test, (samples compared to control infection, *n* = 9, *** *p* < 0.001). Average was displayed with SEM.

Figure 2 shows HaCaT cells with no drug (A–E), with 200 ng/mL bradykinin (F–J) or 200 ng/mL EGF (K–O) for 15 min. Figure 2 shows HPV16 infection of control cells (P–T), cells in the presence of 200 ng/mL bradykinin (U–Y) or 200 ng/mL EGF (Z–DD).

Bradykinin and EGF increased HPV16 PsV infection in HaCaT cells. Cells were treated with either 200 ng/mL of bradykinin or 200 ng/mL EGF for 8 h. The percentage of infection was evaluated for cells treated with bradykinin and EGF via flow cytometry (FF). Statistical significance was determined by ANOVA Dunnett’s multiple comparison test (samples were compared to control infection, *n* = 9, ***, *p* < 0.001). Average was displayed with SEM.

Figure 2EE is a graphic representation of averaged filopodia numbers for our bradykinin and EGF data. The average number of filopodia was 14 for cells alone, 45 after addition of virus, 62 with the addition of bradykinin, and 70 in the presence of virus and bradykinin. Statistical significance was observed in the number of filopodia between cells treated with bradykinin and virus alone (Figure 2EE, *, *p* < 0.05) and between cells treated with virus and cells treated with virus and bradykinin simultaneously (Figure 2EE, **, *p* < 0.01). No statistical significance was observed in filopodia numbers between cells treated with bradykinin and cells treated simultaneously with drug and virus. 

The average number of filopodia was 13 for cells alone, 45 after the addition of virus, 31 with the addition of EGF, and 67 in the presence of virus and EGF. There was a significantly higher number of filopodia after virus addition as compared to EGF (Figure 2EE, *, *p* < 0.05). We observed an additive effect in the number of filopodia when EGF and virus were added simultaneously compared to virus or EGF alone (Figure 2EE, **, *p* < 0.01). 

Filopodia count shown is the average for 15 cells from three separate coverslips (*n* = 45). Statistical significance of filopodia numbers for bradykinin and EGF treatments were evaluated using an ANOVA Dunnett’s multiple comparison test. 

Having shown that bradykinin and EGF induce filopodia, we addressed if filopodia-induction would increase PsV infection of HaCaT cells. Control cells and cells treated with drug in the absence of virus had 0% infection (Figure 2FF). Viral infection 34% was increased to 50.8% (a 49% increase) in the presence of bradykinin. Viral infection 18% was increased to 33.8% in the presence of EGF (an 88.7% increase). An ANOVA Dunnett’s test was used to assess statistical significance. We determined that all samples treated with drug and PsV simultaneously compared to cells treated with PsVs alone were statistically significant with *p* < 0.001, ***. Differences in cell alone viral infections was observed as experiments were performed on different days with different viral stocks.

Although the addition of bradykinin and virus did not have an additive effect on the filopodia number, there was a significant increase in infection when both were added. Our data show an additive effect in filopodia numbers when EGF and virus are added and this resulted in a statistically significant increase in infection. 

**Figure 3:**
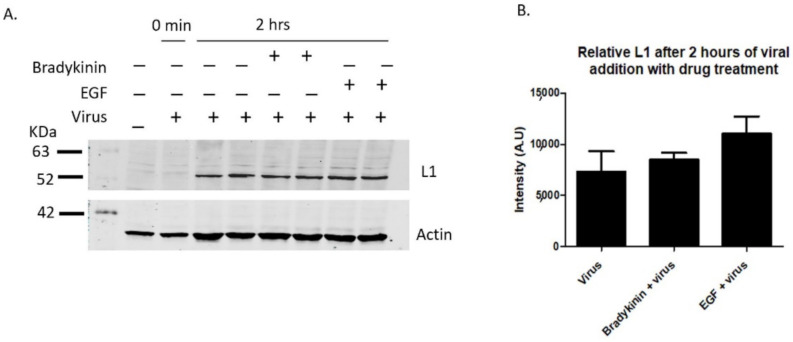
Bradykinin and EGF treatments show increased viral binding. L1 viral capsid protein was measured via western blot after the incubation of 200 ng/mL bradykinin and EGF. PsVs were added for 0 min or 2 h with samples incubated with or without drug on ice. HaCaT cells were washed three times with 1× PBS and then harvested with trypsin. (**A**, 1st lane) control sample without PsVs and drug, (**A**, 2nd lane) control sample with virus that were immediately washed off, (**A**, 3rd and 4th lane) control infection after 2 h, (**A**, 5th and 6th lane) cells treated with bradykinin and virus for 2 h, (**A**, 7th and 8th lane) cells treated with EGF and virus for 2 h. (**B**) Densitometry data of western blot in A showing the relative intensity of L1 protein normalized to actin for two samples from different wells for each treatment with either virus or virus and drug.

Having shown that bradykinin and EGF increase the number of filopodia along HaCaT cell membranes, we next wanted to see if inducing filopodia with the drug would increase overall virus binding. Lane 2 shows a control sample with virus immediately washed off, showing washes removed unbound virus from HaCaT cells (Figure 3A, lane 2, 0 min). When HaCaT cells were treated with bradykinin and EGF simultaneously with virus for 2 h, there was a 15.7% and 50% increase in L1 bound particles, respectively, compared to samples treated with virus alone (Figure 3A,B). 

Our data show that there is a correlation between the increase in filopodia numbers when cells are treated with bradykinin and EGF and an increase in the level of viral binding.

### 3.3. Reducing Filopodia Formation Decreases HPV16 PsV Infection

Having shown that an increase in filopodia numbers resulted in an increase in HPV16 PsV infection in HaCaT cells, we assessed if inhibiting filopodia would decrease infection. We used ML-141, an inhibitor of filopodia formation [19]. 

We tested if ML-141 can inhibit filopodia in HaCaT cells, since to our knowledge this drug has not been previously used in HaCaT cells for filopodia inhibition. We tested if 2 µM, 5 µM, 10 µM, 20 µM, and 40 µM were capable of inhibiting actin protrusions and evaluated the toxicity of these drug treatments. We found that 10 µM ML-141 was a non-toxic concentration that inhibited filopodia (data not shown). We determined that the effect of ML-141 was ineffective after 4 h of drug treatment as filopodia were formed (visualized with confocal microscopy, data not shown). 

**Figure 4:**
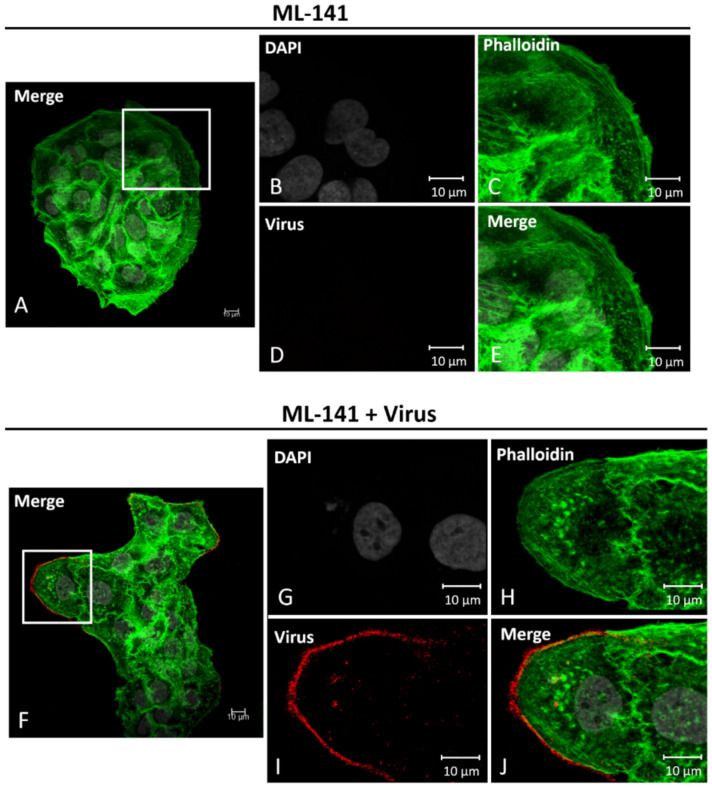

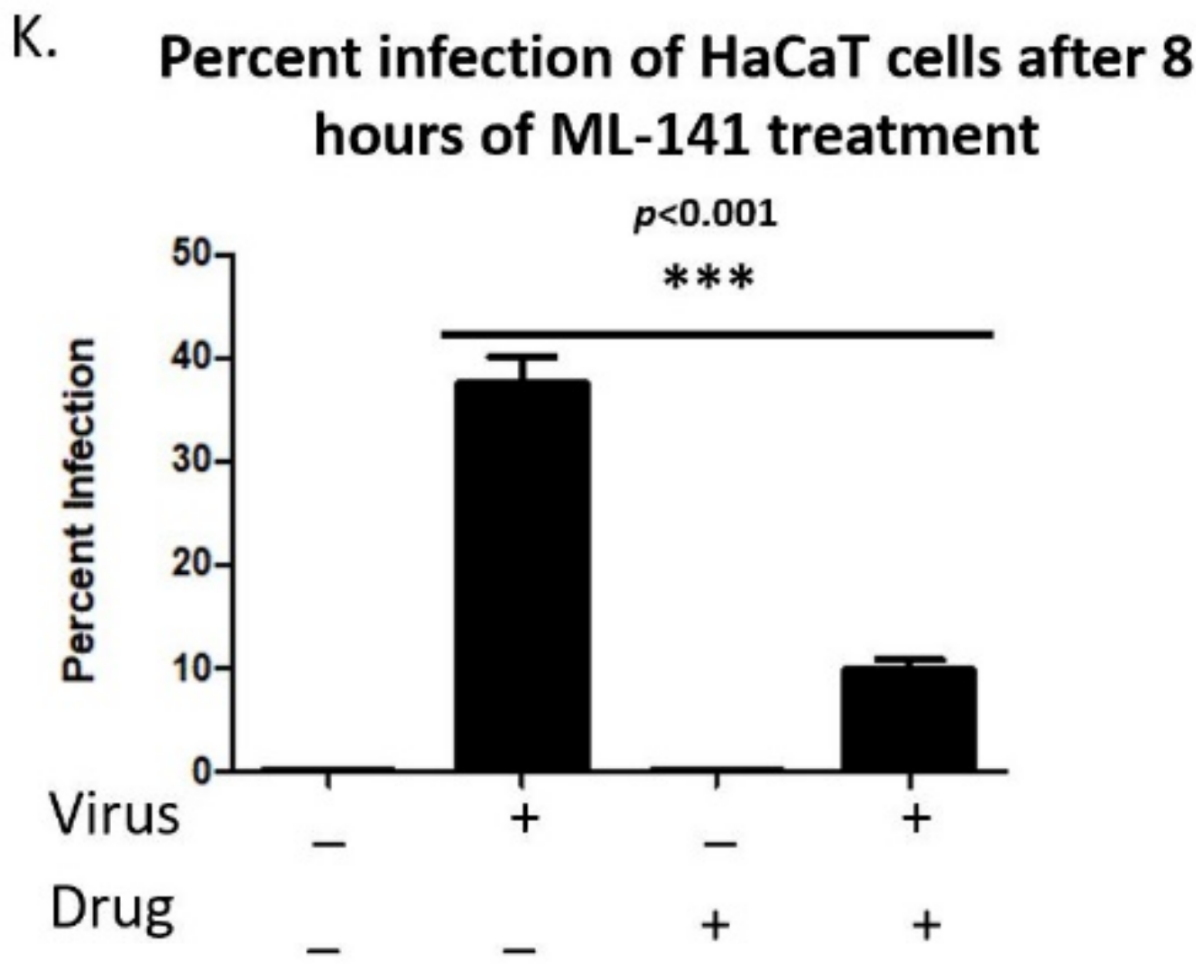
ML-141 treatments inhibit filopodia and decrease HPV16 infection in HaCaT cells. Confocal microscopy images of cells treated with 10 µM ML-141 (**A**–**E**) or treated with both drug and virus (**F**–**J**) for 2 h at 37 °C. Nuclei stained with DAPI (grey), F-actin visualized with phalloidin (green), and L1 capsid protein stained with H16.V5 (red). Merged images of all channels (**E**,**J**). Virus binds along the outside of the HaCaT cell membranes that lack filopodia (**H**). Percent infection with or without drug treatment was measured with flow cytometry (**K**). Data was taken from three individual experiments in triplicate. Statistical differences determined by ANOVA Dunnett’s multiple comparison test, (samples compared to control infection, *n* = 9, *** *p* < 0.001). Average was displayed with SEM.

In Figure 4, HaCaT cells treated with 10 µM ML-141 for 2 h (A–E) and cells treated with ML-141 and PsVs for 2 h (F–J) are shown. These images show that after 2 h of 10 µM ML-141 treatment, filopodia were inhibited even when viruses were simultaneously added (compare Figure 4E,J). 

The infection level of 37.56% decreased to 9.83% in the presence of ML-141 (Figure 4K, 74% decrease, ***, *p* < 0.001). ML-141 drug dilutions were replaced every two hours, thus at 8 h in the experiment there were no filopodia present at 8 h. Lack of filopodia after ML-141 treatment for 8 h was confirmed with immunofluorescence (not shown). Statistical significance was assessed with ANOVA Dunnett’s Multiple comparison’s test, ***p < 0.001, Figure 4K. 

**Figure 5:**
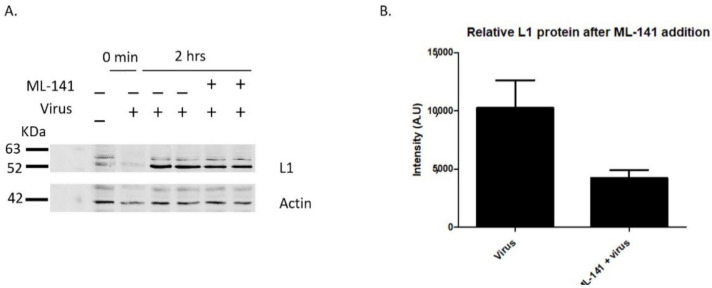
ML-141 treatment reduces viral binding. L1 viral capsid protein was measured via western blot after the incubation of 10 µM ML-141. HaCaT cells were treated with ML-141 for 2 h prior to viral addition at 37 °C. PsVs were added for 0 min or 2 h with samples incubated with or without the drug on ice. HaCaT cells were washed three times with 1× PBS and then harvested with trypsin. (**A**, 1st lane) control sample without PsVs and drug, (**A**, 2nd lane) control sample with virus that was immediately washed off, (**A**, 3rd and 4th lane) control infection after 2 h, (**A**, 5th and 6th lane) cells treated with ML-141 and virus for 2 h. (**B**) Densitometry data of western blot in A showing the relative intensity of L1 protein normalized to actin for two samples from different wells treated with either virus or virus and 10 µM ML-141.

ML-141 treatments did not prevent viral binding (Figure 4J), although there is a 58% reduction in the level of viral particles binding (Figure 5A,B). Lane 2 in Figure 5A shows a control sample with virus that was immediately washed off, showing that PBS washes were effective in removing unbound virus from HaCaT cells (Figure 5A, lane 2, 0 min). Reduction in the level of viral particles binding after ML-141 treatment correlates to the reduction in the number of filopodia, and more work needs to be done to determine if ML-141 interferes with virus binding to the plasma membrane receptor.

Our data demonstrate that inhibiting filopodia significantly decreases infection in HaCaT cells. 

### 3.4. Bradykinin and EGF Treatments Increase Virus Colocalization with Early Endosome Marker and PsVs

**Figure 6:**
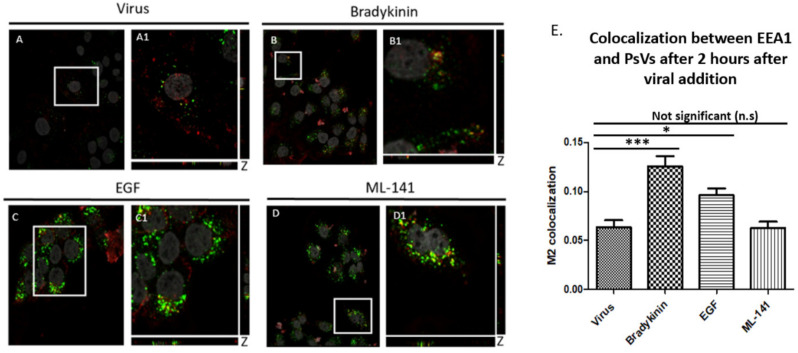
Filopodia inducer drugs increase internalization of PsVs into HaCaT cells. Colocalization measured the amount of PsVs (red) that was trafficked to the early endosome (green). Confocal microscopy images of cells treated with virus and drug for 2 h. Cells treated with virus (**A**,**A1**), 200 ng/mL bradykinin (**B**,**B1**), 200 ng/mL EGF (**C**,**C1**), and 10 µM ML-141 for 2 h (**D**,**D1**). DAPI was used to stain cell nuclei (grey), H16.V5 antibody was used to stain L1 capsid protein (red), and EEA1 was used to stain for early endosome (green). Zoomed in images (**A1**–**D1**). Colocalization of PsVs and EEA1 appeared yellow. The JACoP plugin for ImageJ was used to measure the M2 coefficient (fraction of red overlapping with green) with six confocal Z-scans for each condition. Graph representation of colocalization of six confocal scans (**E**). Statistical difference was determined by an ANOVA Dunnett’s multiple comparison test (samples compared to control infection, *n* = 6, *, *p* < 0.5, ***, *p* < 0.001). Average was displayed with SEM.

Having shown that filopodia inducers increase viral infection and filopodia inhibitors decrease infection, we assessed if these drugs affected internalization and initial trafficking of PsVs into an endosome (Figure 6). 

We quantified virus internalization by measuring the overlap between PsVs (red) and EEA1 [early endosome marker (green)]. Cells were incubated for 2 h with virus (Figure 6A,A1) and: 200 ng/mL bradykinin (Figure 6B,B1); 200 ng/mL EGF (Figure 6C,C1); or 10 µM ML-141 (Figure 6D,D1). 

Figure 6E is the graph representation of the M2 coefficient colocalization data. HPV16 PsV colocalization with EEA1 increased 97% when treated with 200 ng/mL bradykinin (M2 coefficient = 0.1256) compared to cells incubated with virus alone (M2 coefficient = 0.063). We observed a 51.2% increase in colocalization between cells treated with 200 ng/mL EGF (M2 coefficient = 0.096) and cells treated with virus alone. There was only a slight decrease in colocalization of the virus and EEA1 in samples treated with ML-141 (M2 coefficient = 0.062). 

The colocalization averages were evaluated from 15 cells for three separate experiments (*n* = 45). An ANOVA Dunnett’s Multiple comparison’s test was used to evaluate statistical significance. Our data showed that there is an increase in virus internalization when filopodia inducers were added simultaneously with the virus. There was no drastic difference in entry when filopodia are inhibited.

### 3.5. Cdc42 Increases in Activity after Virus Binding

**Figure 7:**
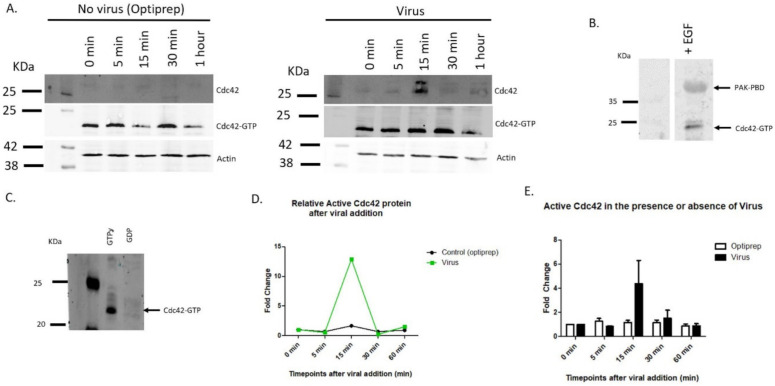
HPV16 activates Cdc42 protein at specific timepoints after viral addition. Active Cdc42 was evaluated with a pull-down using PAK-PBD coated beads. (**A**) Western blot of samples treated with (right side) or without (left side) virus for a single experiment. Blots for Cdc42 total protein, active Cdc42 (GTP bound), and actin. Levels of active Cdc42 was determined for cells incubated with virus for 5 min, 15 min, 30 min, and 60 min. (**B**) Positive control showing Cdc42 activation induced by 200 ng/mL EGF for 15 min. (**C**) Validity of PAK-PBD coated beads were evaluated with control lysates treated with GTPỿ and GDP for 15 min. (**D**) Densitometry data for one individual activation pulldown of Cdc42 with PAK-PBD glutathione beads represented in A. Relative active Cdc42 protein was normalized to 0-min timepoint. A peak in Cdc42 activity after 15 min of viral addition. (**E**) Mean values of seven separate activation pulldown experiments for GTP bound Cdc42. Activation of Cdc42 after viral addition occurs most often between 15–30 min.

Cdc42 has been shown to be involved in filopodia formation, and mediates EGF, bradykinin, and ML-141 signaling [14,18,20]. We evaluated active Cdc42 protein levels after viral addition for 0 min, 5 min, 15 min, 30 min, and 60 min. We observed Cdc42 migrate as a doublet after viral addition (Figure 7A). EGF induced samples were used as positive controls for Cdc42 activation (Figure 7B). PAK-PBD coated beads had a higher affinity to pull-down active GTP bound Cdc42 compared to inactive GDP bound Cdc42 (Figure 6C).

Our data show that active Cdc42 protein levels increased to 13-fold after the addition of virus within 30 min and decreased back to basal levels (Figure 7A,D).

Cdc42 active levels returned to initial levels after 30 min post virus addition. Figure 7E shows mean values of six separate pulldown experiments for active Cdc42 after viral addition. Cdc42 was activated the most between 15–30 min after viral addition (Figure 7E). 

### 3.6. Cdc42 siRNA Decereases HPV16 Infection in HaCaT Cells

**Figure 8:**
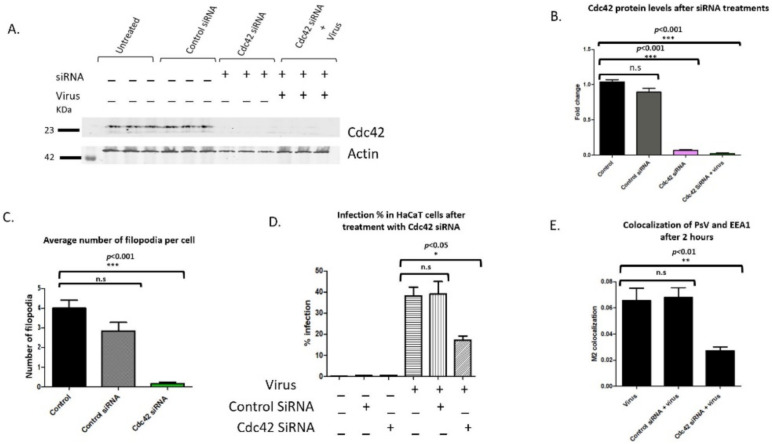
siRNA mediated knockdown of Cdc42 results in a significant decrease in infection and internalization. Cc42 protein levels were knocked down with siRNA. Cells were treated with control siRNA and Cdc42 siRNA for 48 h. (**A**) Western blot analysis of siRNA knockdown using Cdc42 and actin antibodies. Cells were incubated with virus on ice for 2 h and unbound virus was washed from wells. Cdc42 protein levels were normalized with actin. (**B**) Densitometry of western blot showing a significant decrease in Cdc42 protein levels after 48 h even with the addition of virus (samples compared to control with no siRNA or virus, *n* = 3, ***, *p* < 0.001). (**C**). Average number of filopodia per cell after siRNA treatments. Filopodia were counted along the cell periphery with FiloQuant image J plugin, single image analysis. Statistical significance was determined by Dunnett’s multiple comparison test (compared samples to control cells not treated with universal or Cdc42 siRNA, *n* = 50, ***, *p* < 0.001). (**D**) Flow cytometry was performed on cells transfected with control and Cdc42 siRNA. Graph of infection percentages in HaCaT cells treated with control siRNA, Cdc42 siRNA, and virus. Statistical differences were determined by Dunnett’s multiple comparison test (samples compared to control infection, *n* = 9, *, *p* < 0.05). (**E**) Graph representation of colocalization between PsV and EEA1 after 2 h of viral addition for three independent experiments. Statistical difference was determined by unpaired two-tailed *t*-test (*n* = 6, **, *p* < 0.01). Average was displayed with SEM.

Having shown the increase in Cdc42 active protein after viral addition, we next addressed if decreasing Cdc42 protein levels would lower the number of filopodia and affect HPV16 infection in HaCaT cells. siRNA to Cdc42 resulted in a 93% decrease after 48 h (Figure 8A,B). Cdc42 siRNA treated cells had no increase in Cdc42 protein expression after the addition of virus (Figure 8A, last three samples). 

The number of filopodia significantly decreased along the periphery of cells after Cdc42 siRNA treatments compared to control cells and cells treated with control siRNA [*n* = 50, ***, *p* < 0.001, Dunnett’s multiple comparison test, (Figure 8C)]. We confirmed siRNA mediated knockdown of Cdc42 and the resulting depletion of filopodia with confocal microscopy (Supplementary Figure S4A–J). 

There was a 55% decrease in infection when cells were treated with Cdc42 siRNA for 48 h compared to control infection [17.2% to 38.15%, respectively (Figure 8D)]. Statistical significance was evaluated for three separate experiments (*n* = 9, *, *p* < 0.05, Dunnett’s multiple comparison test was used to measure the statistical difference between samples and control infection). 

We further evaluated virus internalization by observing the overlap between PsV and EEA1. There was a statistically significant decrease of 59% in colocalization of cells treated with Cdc42 siRNA compared to the control infection (Figure 8E). Dunnett’s multiple comparison test was used to measure statistical difference between samples and the control infection (*n*= 6, *, *p* < 0.01). 

Our data shows that Cdc42 depleted cells had significantly less filopodia, lower infection compared to control infection, and decreased overlap between PsVs and EEA1. Taken together, our data suggest a role for active Cdc42 in HPV16 infection through its role in filopodia formation.

#### Cdc42 Knockdown Cells Have Lower Infection and Are Arrested at the G1 Phase

Having shown that infection decreased in cells treated with Cdc42 siRNA, we investigated whether this change in infection was due to cell cycle arrest due to the loss of Cdc42 protein. Cdc42 GTPase has a well-described role in promoting G1 cell cycle progression [21]. 

**Figure 9:**
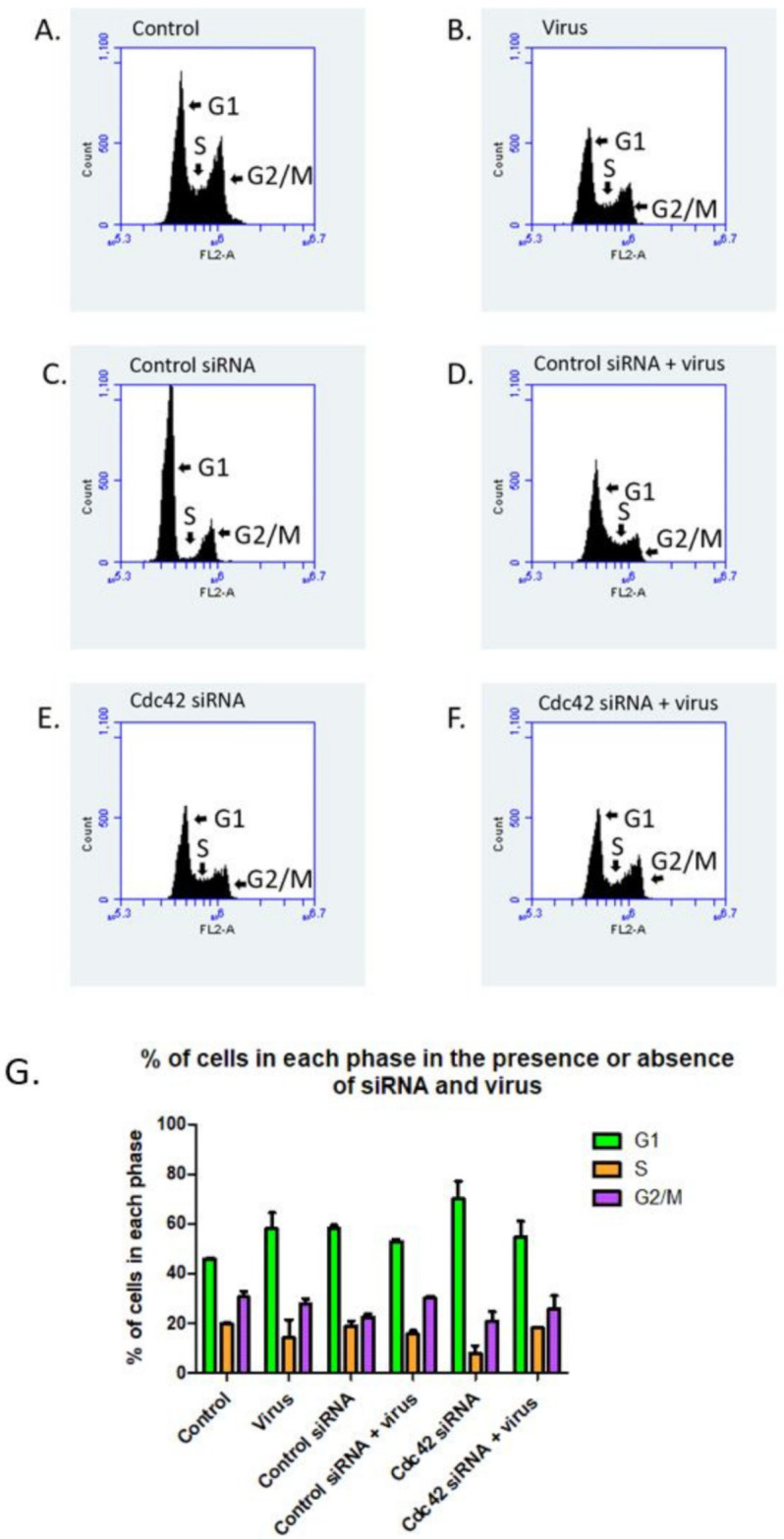
Cells treated with Cdc42 siRNA undergo cell cycle arrest in the presence and absence of virus. Histograms showing the percentage of cells in different stages of the cell cycle based on DNA content examined by propidium iodine staining via flow cytometry (**A**–**F**). Control cells not treated with virus or siRNA (**A**), cells treated with virus (**B**), cells treated with control siRNA (**C**), cells treated with both control siRNA and virus (**D**), cells treated with Cdc42 siRNA (**E**), and cells treated with both Cdc42 siRNA and virus (**F**). Graphic representation of the percentage of cells in each stage of the cell cycle after each treatment (**G**), *n* = 3. Cells were treated with control or Cdc42 siRNA for 48 h prior to virus addition. Virus was permitted to bind to cells for 2 h on ice with remaining virus removed with PBS washes. Flow cytometry was performed 48 h after viral addition.

We analyzed the DNA content of HaCaT cells treated with or without control siRNA or siRNA against Cdc42 in the absence or presence of virus using flow cytometry (Figure 9A–F). Control cells and cells treated with control siRNA show comparable cell cycle profiles in G1 (45.95% and 52.92%, respectively), S (21.29% and 18.82%, respectively) and G2 phase [30.5% and 22.5%, respectively, (Figure 9A,C). 

We confirmed that Cdc42 siRNA transfection inhibited cell cycle progression with a 53% increase in cells at the G1 phase compared to control cells. There was a 64% decrease of cells in the S phase and a 47% decrease of cells in G2/M phase in Cdc42 knockdown cells compared to control cells (compare Figure 9A,E). 

It is interesting that when we compare infected cells treated with Cdc42 siRNA and uninfected cells treated with Cdc42 siRNA, there is a greater population of cells in the S phase (18% and 7.59%, respectively) and in the G2/M phase (25.7% and 20.76%, respectively). 

## 4. Discussion

HPV16′s use of non-traditional endocytosis mechanisms to enter epithelial cells remains unclear. Studies have shown that HPV16 does not utilize the key molecules involved in clathrin, caveolin, and flotillin based modes of entry [5,22]. One common process involved in HPV16 entry is the requirement for host actin rearrangement [4,17,22]. Filopodia are membrane extensions that are formed during specific actin rearrangement events. In this manuscript we show that there is a direct relationship between the number of filopodia and level of infection.

We had previously observed an increase in filopodia on HaCaT cell membranes after the addition of virus [8]. In that original manuscript we had shown that in cells treated with an inhibitor FAK, a kinase involved in cytoskeletal rearrangements leading to filopodia formation, virus infection was prevented [7]. Here we specifically designed experiments to assess if increases or decreases in filopodia numbers affected infection, and if HPV16 binding was responsible for inducing the de-novo formation of filopodia. We show that Cdc42 activity is partially influencing the formation of filopodia and initial infection.

We hypothesized that there were two major ways HPV16 interacted with filopodia. First, HPV16 virions are immobilized in the extracellular matrix (ECM) and come in contact with migrating cells or elongating filopodia from nearby cells. The virus interacts with a plasma membrane receptor that is either along the filopodia structure or at a different location along the cell membrane and is endocytosed. Second, virus-receptor interactions at the plasma membrane resulted in a signaling cascade that increased the number of filopodia and infection.

Our experiments specifically show that, as previously described, the addition of viral particles onto cells in tissue culture results in initial binding of virions to the ECM and to the cell membrane. Our analysis shows that the binding in the ECM overlaps with the presence of laminin-5. We theorized that if there were more filopodia present, there would be an increase in the infection process. We compared the level of infection in conditions in which we artificially increased or decreased the number of filopodia. We interpreted virus internalization two ways: (1) GFP expression of viral pseudogenome using flow cytometry after HaCaT cells were infected with PsVs for 48 h, and (2) colocalization of the virus with the early endosome using immunofluorescence. 

Our data showed that cells treated with bradykinin alone had significantly more filopodia compared to cells treated with virus. Although the number of filopodia between cells treated simultaneously with both bradykinin and virus had a significant increase compared to cells treated with virus alone, the addition of both drug and virus did not have an additive effect. Infection levels was additive and increased by 49% in cells treated with bradykinin and virus compared to the control infection. 

Our data for our EGF experiments differ from those performed with bradykinin. Cells treated with EGF alone had significantly less filopodia compared to cells treated with virus. Cells treated with both EGF and virus had an additive effect to the number of filopodia, and there was an additive 88% increase in infection when the drug was added with virus simultaneously. These data indicate bradykinin and EGF may use different mechanisms to increase filopodia numbers. It is notable that infection levels in the presence of bradykinin or EGF were higher than in the presence of virus alone. 

We hypothesize that bradykinin and EGF treatments increase infection by inducing filopodia, which results in increased viral binding. Filopodia increase the surface area of the cell membrane and offer opportunities for viruses to encounter their uptake receptors or endocytic machinery. We showed that cells treated with bradykinin had a 15.7% increase and EGF treated cells had a 50% increase in bound L1 particles compared to cells treated with virus alone. Viruses can bind to attachment receptors along filopodia structures that can act as entry factors. 

We investigated if the presence of filopodia is connected to earlier viral entry by measuring virus internalization after the addition of bradykinin and EGF for 2 h. Early endosome was used as a marker for virus internalization because it is one of the first organelles the virus is trafficked to after entry. Cells treated with bradykinin showed a 97% increase in colocalization between EEA1 and PsVs. Cells treated with EGF had a 51.2% increase in colocalization. Our data supports our original hypothesis that if infection was increased in cells treated with drug and virus then the overlap between PsVs and EEA1 would be increased as well.

Our data show that in the absence of filopodia, we observe less total binding of viral particles. These results are comparable to the Cdc42 siRNA infection and colocalization data. Viral infection and PsV colocalization with EEA1 were significantly decreased when cells were treated with Cdc42 siRNA 48 h prior to viral addition. We did observe a greater decrease in infection when cells were treated with ML-141 (74%) compared to cells treated with Cdc42 siRNA (55%). However, filopodia inhibition with ML-141 did not prevent viruses from entering cells in the first 2 h after viral addition. The colocalization coefficient between ML-141 treated cells were similar to cells treated with virus alone. Cells pretreated with Cdc42 siRNA had a 59% decrease in colocalization. Using the inhibitor of filopodia, ML-141 did not result in a complete loss of infection, supporting the hypothesis that virus binds directly to a plasma membrane receptor independent of filopodia present. 

Our data show that HPV16 addition results in filopodia induction. The data suggest that HPV16 binds to a membrane receptor and a signal transduction event is activated. We investigated Cdc42 GTPase, a well-studied signaling protein that regulates filopodia formation and actin rearrangement [23,24]. Cdc42 plays a role in the infectious process for several viruses, including HIV-1, HSV-1, EHV, EBV, HHV-8, strains of VACV, DEV-2, and RSV [25,26,27,28,29,30]. Using a PAK-PBD pulldown assay for active Cdc42, we demonstrate that there is an increase in GTP bound Cdc42 after 15 min of virus addition in HaCaT cells. In other experiments we have also observed an increase in Cdc42 proteins at 30 min after viral addition. In our activation assays we saw Cdc42 protein migrated as a doublet, and more work needs to be done to explain why we observe this at the timepoints after viral addition. We hypothesize that the virus binds to a receptor which leads to cell signaling events that activate Cdc42. We have seen Cdc42 activated at 15 and 30 min with a relationship of increased filopodia formation at both of these timepoints. The activation of Cdc42 and filopodia formation may be in a range between 15–30 min. Our data suggest that there is a transient activation decreasing after 1 h. 

We demonstrate Cdc42′s importance in HPV16 infection by decreasing the Cdc42 protein. SiRNA mediated knockdown of Cdc42 inhibited filopodia formation and drastically decreased HPV16 infection in HaCaT cells. HSV-1 shows similar reliance on Cdc42 for actin rearrangement during infection. HSV-1 activates Cdc42 in the first 30 min of virus exposure to induce signaling cascades responsible in a phagocytotic internalization [31]. The VAV-ihdj strain was shown to have a similar increase in Cdc42 activity after 15 min of virus addition, and activity lasted for 60 min. VAV-ihdj virions utilized filopodia for viral surfing and macropinocytosis entry [32]. We still saw infection and overlap of virus with EEA1 after ML-141 inhibition of Cdc42 and siRNA targeted treatments, albeit significantly decreased, which suggests that Cdc42 may not be essential for viral entry into cells but potentially in viral trafficking. 

Infection by papillomaviruses is believed to be enhanced during the process of wound healing. Would healing is meant to close the gap in the keratinocyte layer due to a physical tear. Our data supports the finding of Dr. Ozbun’s lab showing that HPV31 (in the ECM) infects HaCaT cells by binding to the filopodia of migrating cells [33]. 

Our data clearly showed a higher level of infection in the presence of filopodia vs. in the absence of filopodia (compare bradykinin/EGF experiments with ML-141). This finding is similar to Dr. Shukla’s findings using HSV-1 in P19 neural cells, i.e., they show HSV-1 induces filopodia to increase efficiency of viral infection [34]. Filopodia are rich with heparan sulfate proteoglycans (HSPGs), glycoproteins found along the cell membrane, and other receptors in order to perform their innate roles in environment probing and cell-cell interactions [35]. HSPGs are common binding receptors for HSV-1 and HPVs, thus suggesting that there is a higher availability of receptors [36]. More work needs to be performed to determine if our higher number of filopodia correlate with a higher level of viral receptors, e.g., heparan sulfate proteoglycans. 

The number of filopodia had a direct effect on the number of internalized viral particles in our experiment. The number of overlapping HPV16 virions with EEA1 was higher in bradykinin and EGF than in the presence of ML-141. We indeed see internalization in the absence of visible filopodia (i.e., ML141 experiments) suggesting that filopodia are not required for endocytosis. Similar findings were shown by Schelhaas et al., who reported that filopodia inhibition and actin depolymerization drugs did not have an effect on viral infection in confluent HaCaTs cells. As Schelhaas et.al. indicated in their article, infection may still occur after actin depolymerization and filopodia inhibition because viruses may directly bind to the cell body instead of filopodia [4]. 

Filopodia are exploited by viruses to enter target cells. Viruses can bind to cell membranes and stimulate signaling pathways that induce filopodia formation and macropinocytosis. The vaccinia virus, an ihd-j MV strain, has been shown to induce rapid lengthening of filopodia which result in macropinocytosis. The filopodia lengthen and then fold back in towards the membrane. HPV16 is suspected to use a macropinocytosis-like mechanism; however, it is distinct because it does not use traditional signaling molecules associated with this process [5]. Filopodia can also be used to increase the efficiency of viral transmission to neighboring uninfected cells. Retroviruses have been shown to infect cells two to three orders higher when viruses established filopodial bridges to uninfected cells [37]. It is possible that HPV16 virions are moved in a similar way to retroviruses by first interacting with cells bordering the wound and then moving to uninfected cells as the gap is closed and new cell-cell attachments are formed.

Viruses can use filopodia to move horizontally or laterally along the membrane in the process of actin-retrograde movement or viral surfing. In one study, HPV16 was observed moving in a directed motion along actin protrusions in HeLa cells in a comparable speed to the movement of EGFR receptors along membranes during actin-retrograde movement [4]. When F-actin disrupting drugs were used, such as blebbistatin and Cytochalasin D, HPV16 movement along the membrane was random. Viral surfing involves the lateral movement of viruses along the membrane. Studies have suggested that viral surfing allows viruses to interact with receptors present along filopodia. Eventually viruses come in contact with receptors that induce signaling involved in viral entry. HSV-1 has been shown to interact with heparan sulfate and gB receptors present in higher densities along filopodia, which allowed for viral surfing and eventual internalization [30]. It is most likely that HPV16 exploits filopodia in multiple ways to gain access to the membrane receptors during entry. 

In this study, we have shown that HPV16 binding to a cell membrane receptor induces filopodia. Our results suggest that an increase in filopodia numbers increases infection. We show that bradykinin and EGF increases internalization by 97% and 51.2%, respectively. We have shown that a filopodia inhibitor did not decrease early entry of the virus. Taken together, filopodia help increase the efficacy of viral entry but they are not required; similar claims were found by Schelhaas et al. [4]. We have investigated Cdc42 as a potential molecule involved in HPV16-induced filopodia signaling. When Cdc42 protein is knocked down or the ML-141 allosteric inhibitor is used, there is a significant decrease in infection, suggesting that Cdc42 may play a role in trafficking.

HPV16 relies on mitotically active epithelial cells [38]. We investigated if the observed loss of infection was a result of Cdc42 knockdown due to Cdc42s’ role in cell cycle progression [39,40]. Our data showed a loss of cell cycle progression in the presence of Cdc42 siRNA; thus, the loss of infection can be in part attributed to cell cycle arrest in addition to Cdc42’s role in filopodia formation. Taken together, additional studies done on upstream and downstream proteins of Cdc42 signaling will provide valuable information on HPV16 entry.

## 5. Conclusions

HPV16 utilizes non-traditional mechanisms that are dependent on actin rearrangement to enter cells. Here we investigated the process of HPV16-induced filopodia formed as a result of actin rearrangement. Our data shows that when filopodia formation was initiated by drugs there was a highly significant increase in infection; and conversely, when filopodia were inhibited, there was a significant decrease in infection. When cells are treated with filopodia inducers simultaneously with virus we observed an increase in virus internalization compared to infection without the drug. To further evaluate filopodia’s role in HPV16 infection we targeted Cdc42 GTPase, a well-known filopodia signaling molecule. We observed a significant decrease in infection when both siRNA and drugs against Cdc42 were used in HaCaT cells. Our data suggest that HPV16 infection is not dependent on filopodia formation, but these structures may help with early entry into target cells. 

## Data Availability

Not applicable.

## Supplementary Materials

The following supporting information can be downloaded at: https://zenodo.org/record/6400121#.Yo8Nx1RByUk: Figure S1. FiloQuant analysis of filopodia; https://zenodo.org/record/6400130#.Yo8NzFRByUk: Figure S2. Laminin-5 is localized in areas with high filopodia density; https://zenodo.org/record/6400133#.Yo8Nz1RByUk: Figure S3. Filopodia numbers decrease along HaCaT cell membranes at later timepoints of infection; https://zenodo.org/record/6400135#.Yo8N0lRByUk: Figure S4. Optimal conditions for Cdc42 siRNA mediated knockdown.
